# Accuracy of Static Computer-Aided Implant Surgery: A Clinical Comparison of Tooth-, Bone-, and Mucosa-Supported Surgical Guides

**DOI:** 10.3390/jfb17040194

**Published:** 2026-04-17

**Authors:** Igor Smojver, Roko Bjelica, Marko Vuletić, Luka Stojić, Vlatka Njari Galić, Dragana Gabrić

**Affiliations:** 1 Department of Oral Surgery, School of Dental Medicine, University of Zagreb, 10000 Zagreb, Croatia; ismojver@sfzg.hr (I.S.); mvuletic@sfzg.hr (M.V.); dgabric@sfzg.hr (D.G.); 2 St. Catherine Specialty Hospital, 10000 Zagreb, Croatia; 3 Department of Dental Medicine, Clinical Hospital Centre Zagreb, 10000 Zagreb, Croatia; 4 Private Practice DentA Centar, 10000 Zagreb, Croatia; lukastojic@gmail.com; 5 Mund-Kiefer-Gesichtschirurgie Steffen Wirth, 82538 Geretsried, Germany; vnjari1@gmail.com

**Keywords:** surgery, computer-assisted, dental implants, jaw, edentulous, printing, three-dimensional

## Abstract

The accuracy of static computer-aided implant surgery (s-CAIS) is fundamental for predictable clinical outcomes. The objective of this study was to evaluate the influence of different guide-support modalities on the linear and angular accuracy of implant placement. In this retrospective clinical investigation conducted at a single specialty hospital, a total of 180 implants were analyzed, divided into three equal groups (n = 60) based on the guide support type: tooth-supported, bone-supported, and mucosa-supported. Accuracy was assessed by superimposing preoperative virtual plans with postoperative cone-beam computed tomography (CBCT) scans, measuring linear deviations at the neck and apex of the implant, as well as angular discrepancies. The type of guide support was found to be a significant factor associated with surgical accuracy (*p* < 0.001). Tooth-supported guides demonstrated the highest level of accuracy, with a mean angular deviation of 1.81° ± 0.45° and linear deviations at the neck and apex of 0.59 ± 0.18 mm and 0.73 ± 0.19 mm, respectively. These were followed by bone-supported guides (2.14° ± 0.48°; 1.04 ± 0.26 mm; 1.61 ± 0.31 mm), while mucosa-supported guides exhibited the greatest deviations (2.95° ± 0.60°; 1.47 ± 0.29 mm; 1.87 ± 0.37 mm). Significant intergroup differences and large effect sizes were observed, particularly regarding angular and horizontal discrepancies. These findings demonstrate a distinct gradient of accuracy based on guide support, establishing tooth-supported guides as the most accurate, followed by bone-supported and, lastly, mucosa-supported guides. While all modalities are clinically applicable, the use of mucosa-supported guides necessitates increased safety margins to account for the increased risk of linear and angular discrepancies inherent to mucosal tissue displacement.

## 1. Introduction

The paradigm shift in dental implantology from a purely surgical approach to a prosthetically driven digital workflow has revolutionized treatment predictability. Within this framework, static computer-aided implant surgery (s-CAIS) has become a vital tool, which utilizes virtual planning based on the integration of cone-beam computed tomography (CBCT) and intraoral surface scans [1]. This allows for a meticulous preoperative assessment of bone volume, vital anatomical structures, and the desired prosthetic position. Consequently, s-CAIS has been associated with numerous clinical benefits, including minimally invasive surgical procedures, reduced duration of surgery, reduced postoperative discomfort, and the optimization of biomechanical loading through precise implant positioning [2].

However, the transition from a virtual three-dimensional plan to the actual clinical execution is inherently subject to various inaccuracies [3]. The multi-stage nature of s-CAIS inevitably gives an opportunity for errors to occur at any point, starting from image acquisition and data segmentation to the manufacturing tolerances of 3D-printed surgical guides [4]. Furthermore, the clinical result is influenced by registration procedures, mechanical tolerances between the guide sleeve and the drills, the stability of the fixation method, limited surgical access, and the mechanics of the implant insertion process itself. Among the various intraoperative factors influencing the final position of the implant, the stability and rigidity of the surgical guide’s support are considered paramount. Current clinical protocols primarily utilize three types of surgical guides: tooth-supported, bone-supported, and mucosa-supported guides [5]. Each of these modalities interacts differently with the underlying tissues, presenting unique challenges regarding stability and displacement during the osteotomy and implant placement.

Tooth-supported guides are generally regarded as the gold standard for accuracy due to the non-resilient nature of the dental enamel, which provides a rigid and repeatable seat [6]. Nevertheless, their application is limited to partially edentulous patients with a sufficient number of stable teeth. For completely edentulous patients, clinicians must choose between bone-supported and mucosa-supported surgical guides [7]. Mucosa-supported guides offer a significant advantage by enabling flapless surgery; however, they rely on the overlying soft tissue, which is inherently displaceable. The resilience of the oral mucosa can lead to unexpected tilting or “sinking” of the guide under surgical pressure during osteotomy, potentially resulting in significant angular and horizontal deviations [8]. Conversely, bone-supported guides require extensive flap elevation for direct placement on the alveolar bone, providing high rigidity but introducing complexities such as the potential for improper seating due to limited visual verification or anatomical irregularities [9].

The scientific literature has extensively documented these discrepancies, yet a consensus on the definitive superiority of one support type in varied clinical scenarios remains elusive. A meta-analysis by Raico Gallardo et al. [10] highlighted that while guided surgery is superior to free-hand placement, the tissue of support significantly impacts the magnitude of error. More recently, a systematic review by Shi et al. [11] emphasized that mucosa-supported guides consistently exhibit the lowest in vivo accuracy, whereas bilateral tooth-supported systems reach the highest precision levels. The latest evidence underscores the necessity of continuous clinical validation as 3D printing technologies and planning software evolve [12]. Despite these insights, there is a lack of high-powered, standardized clinical trials that directly compare all three support modalities using a uniform surgical and measurement protocol within the same clinical environment [13].

This retrospective study was designed to evaluate and compare the accuracy of implant placement using tooth-supported, bone-supported, and mucosa-supported surgical guides. The main objective was to quantify linear deviations at the implant neck and the apex, along with angular discrepancies. Through this investigation, clinicians could be provided with refined safety margins and evidence-based recommendations for selecting the most appropriate guidance strategy in complex rehabilitations.

## 2. Materials and Methods

### 2.1. Study Design and Ethical Considerations

This retrospective clinical study was conducted between January 2024 and April 2025 at the St. Catherine Specialty Hospital, Zagreb. The study protocol was designed in strict accordance with the Declaration of Helsinki and received formal approval from the Institutional Review Board of St. Catherine Specialty Hospital (protocol code: UR VII/23-4, approved date: 15 December 2023). All participating patients were thoroughly informed about the surgical procedure, potential risks, and the utilization of their anonymized clinical data for research purposes, after which they provided written informed consent. All surgical and digital procedures were performed according to a standardized clinical protocol established at the institution to ensure procedural consistency.

### 2.2. Patient Selection and Sample Size Calculation

The study population consisted of partially and completely edentulous patients who required implant-supported prosthetic rehabilitation in either the maxilla or the mandible. An *a priori* power analysis was performed using G*Power software (version 3.1.9.7, Heinrich-Heine-Universität Düsseldorf, 40225 Düsseldorf, Germany). Based on existing literature regarding accuracy discrepancies in static computer-aided implant surgery (s-CAIS), the calculation was set for a one-way ANOVA across three independent groups with a large effect size (f = 0.40), a significance level of *p* = 0.05, and a statistical power (1 − β) of 0.80. The analysis indicated that a total of 66 implants (22 per group) would be sufficient to detect significant differences. The individual implant was defined as the primary unit of analysis. To further enhance the validity of the findings, the final sample was expanded to 180 implants, equally distributed into three cohorts (n = 60 per group): tooth-supported, bone-supported, and mucosa-supported guides.

Strict eligibility criteria were applied for patient inclusion. Patients were eligible if they were: (1) at least 21 years of age; (2) possessed adequate bone volume allowing for the placement of standard dimension implants without the need for simultaneous guided bone regeneration (Cawood and Howell Class III or IV); and (3) exhibited healthy oral mucosa. Conversely, exclusion criteria included: (1) uncontrolled systemic diseases (ASA III/IV); (2) history of radiotherapy in the head and neck region; (3) active substance abuse or heavy smoking (>10 cigarettes per day); (4) poor oral hygiene or untreated periodontal disease; and (5) pregnancy or lactation at the time of surgery.

### 2.3. Digital Workflow and Preoperative Planning

A standardized digital diagnostic protocol was implemented for all patients. CBCT scan (Orthophos SL, Dentsply Sirona, Bensheim, Germany) was performed to obtain volumetric datasets in Digital Imaging and Communications in Medicine (DICOM) format. The scanning settings were: tube voltage of 85 kV, tube current of 6 mA, exposure time of 14.4 s, and a cylindrical field of view (FOV) of 8 cm x 8 cm. The reconstructed voxel size was 160 µm. For partially edentulous patients, the clinical situation was captured using an intraoral scanner (TRIOS 3 wireless, 3Shape, Copenhagen, Denmark) to generate high-definition surface models in Standard Tessellation Language (STL) format, representing the remaining dentition and mucosal surfaces. In contrast, for completely edentulous patients, clinical prosthetic information was captured indirectly through a radiographic guide as part of the dual-scan protocol, eliminating the need for separate intraoral STL surface scans. All digital datasets were imported into the coDiagnostiX^®^ planning software version 10.6 (Dental Wings Inc., Montreal, QC, Canada) for virtual implant positioning and surgical guide design. The guides were 3D-printed with Straumann P20+ (Institut Straumann AG, Basel, Switzerland) printer using a specialized biocompatible clear resin (P Pro Surgical Guide Clear, Institut Straumann AG, Basel, Switzerland).

#### 2.3.1. Partially Edentulous Patients (Tooth-Supported Guides)

For partially edentulous patients, the integration of digital data relied on the superimposition of STL surface scans onto the CBCT DICOM datasets. This was achieved through a point-to-point registration technique, followed by an automated best-fit algorithm using the stable surfaces of the remaining teeth as reference landmarks. This alignment allowed for a prosthetically driven planning approach, where the implant positions were determined based on the relationship between the underlying bone volume and the planned crown positions. Teeth indicated for extraction were virtually removed in the planning software. For stabilization of the surgical guide, two mesial teeth and the most distal teeth on each side were preserved, enabling a tooth-supported guide design. The surgical guides were designed with a rigid seat on the occlusal and incisal surfaces of the anchor teeth to ensure maximum stability. Specifically, a strict 0.10 mm offset was implemented in the design software to ensure a tight friction fit, allowing the guide to “click” and mechanically lock onto the remaining dentition.

#### 2.3.2. Completely Edentulous Patients (Bone and Mucosa-Supported Guides)

To overcome the lack of fixed dental landmarks in completely edentulous cases, a standardized dual-scan protocol was employed [14]. A radiographic guide was prepared by modifying a well-fitting existing denture with eight spherical radiopaque markers embedded into the denture.

The first CBCT scan was performed with the patient wearing the radiographic guide. A second CBCT scan was subsequently performed on the radiographic guide alone using identical exposure parameters. The datasets were superimposed within the planning software, using radiopaque markers as reference points, creating a “virtual patient” model.

Bone-supported guides were designed to be seated directly on the cortical bone after full-thickness mucoperiosteal flap elevation. Mucosa-supported guides were designed to rest on the keratinized mucosa, following the soft tissue contours captured by radiographic guide.

### 2.4. Surgical Procedure

All surgical procedures were performed by a single specialist of oral surgery to eliminate inter-operator variability. The interventions were conducted under local anesthesia using 4% articaine with 1:100,000 adrenaline (Ubistesin Forte, 3M ESPE, Seefeld, Germany). Prior to the surgery, patients performed a 60 s preoperative rinse with a 0.2% chlorhexidine digluconate solution.

**Figure 1:**
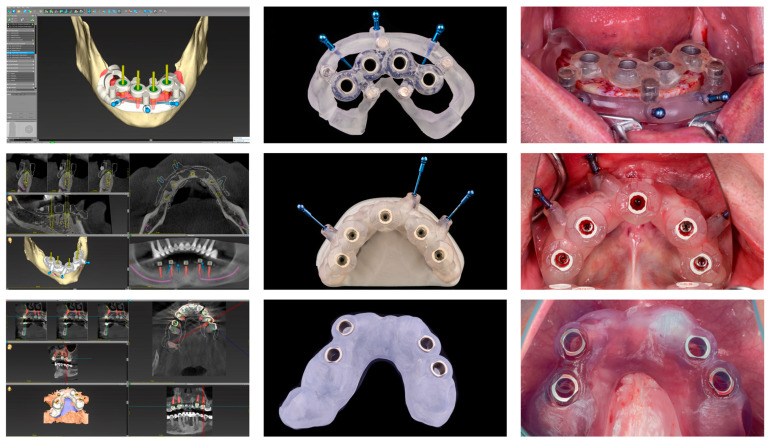
Integrated digital workflow, from initial virtual planning and design of the surgical guides to the final clinical application, for each support modality. (**top row**) Bone-supported guide; (**middle row**) Mucosa-supported guide; (**bottom row**) Tooth-supported guide.

The clinical application of the surgical guides varied according to the specific support modality of each study group (Figure 1):

- Tooth-supported: The surgical guide was positioned onto the remaining dentition. Intraoperative stability was verified visually and mechanically through inspection windows to ensure a precise and intimate fit on the occlusal and incisal surfaces. Due to the 0.10 mm design offset, the guide achieved a stable friction fit. The surgeon’s manual pressure was applied only as a secondary, precautionary measure throughout the osteotomy and implant placement.
- Bone-supported: Initially, a primary (base) guide was utilized to perform pilot drilling for the anchor pins. Following this, a full thickness mucoperiosteal flap was elevated to fully expose the alveolar ridge. The definitive surgical guide was seated directly onto the cortical bone. To ensure a stable and repeatable position, the guide was secured using three or four horizontal anchor pins (Straumann^®^ Guided Surgery, Institut Straumann AG, Basel, Switzerland). These pins provided bi-cortical stabilization, preventing any rotational or linear displacement during osteotomy.
- Mucosa-supported: A flapless surgical approach was utilized. The guide was positioned on the keratinized mucosa and stabilized as planned during the dual-scan protocol. To ensure rigid immobilization and prevent displacement during the procedure, the mucosa-supported guide was also secured using horizontal anchor pins, following a stabilization protocol similar to that of the bone-supported approach.

Following the rigid fixation of the guides, the osteotomy was performed using the Straumann^®^ Guided Surgery system. A fully guided drilling sequence was executed according to the manufacturer’s protocol. The drilling was performed under continuous irrigation with sterile saline. The speed and torque were strictly controlled as per the manufacturer’s recommendations for each bone quality type.

All implants (Straumann^®^ SLActive, Institut Straumann AG, Basel, Switzerland) were inserted “fully guided” through the surgical guide. This ensured that the depth, angulation, and mesiodistal/buccolingual positions were a direct transfer of the virtual plan. Mucoperiosteal flaps were sutured using non-resorbable monofilament sutures in cases where flap elevation was performed.

Postoperative instructions included the use of analgesics (ibuprofen 600 mg) as needed and a 0.2% chlorhexidine rinse twice daily for seven days. All patients received antibiotic therapy (amoxicillin/clavulanic acid 1 g every 12 h) starting one day preoperatively and continuing for five days postoperatively.

### 2.5. Accuracy Assessment

To quantify the discrepancies between the virtual planning and the clinical outcomes, postoperative CBCT scans were obtained for all patients after the implant healing period (3–4 months postoperatively). These scans were performed using the same radiographic unit and standardized acquisition parameters as the preoperative imaging. They were clinically indicated as part of the routine follow-up protocol prior to the prosthetic phase. The imaging procedure followed the As Low as Reasonably Achievable (ALARA) principles.

The postoperative DICOM datasets were imported into the coDiagnostiX^®^ treatment evaluation module. The superimposition of the postoperative data onto the preoperative planning datasets was achieved through an automated surface best-fit matching protocol. This process utilized the Iterative Closest Point (ICP) algorithm, which aligns the datasets by minimizing the distance between stable anatomical landmarks. To ensure the reliability of the measurement protocol, a single experienced examiner repeated all measurements after a two-week interval to assess intra-observer reliability.

**Figure 2:**
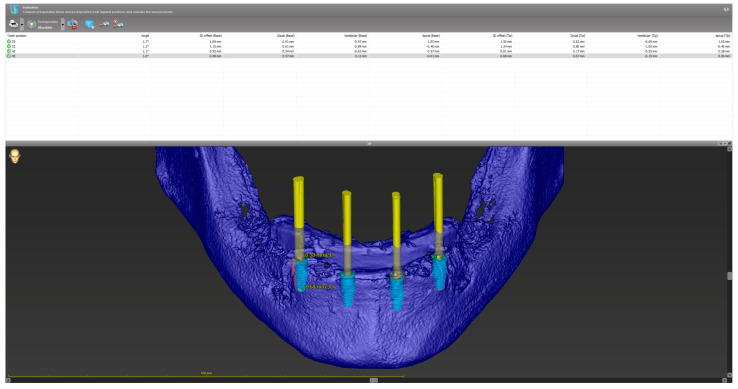
Accuracy evaluation within the treatment evaluation module of coDiagnostiX^®^, showing the 3D superimposition of the planned (blue) and actual (red) implant positions across the entire arch.

**Figure 3:**
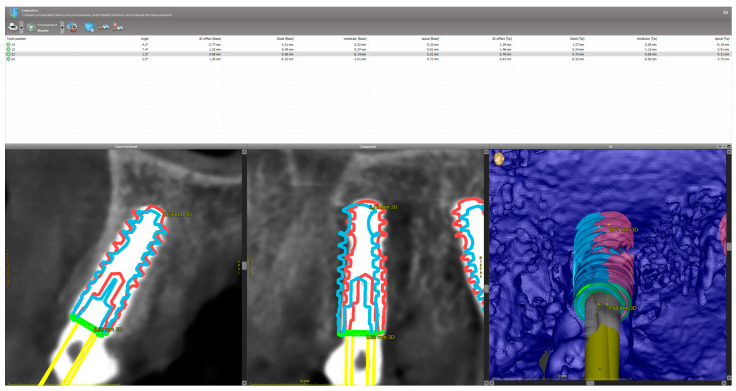
Macro-scale accuracy assessment within the treatment evaluation module of coDiagnostiX^®^, demonstrating detailed 3D superimposition views by comparing the planned (blue) and actual (red) implant trajectories.

Once the virtual models were aligned, the software automatically calculated the following four deviation parameters for each implant by comparing the planned position with the actual position (Figure 2 and Figure 3):

- 3D Deviation at the implant neck: The linear distance (mm) between the centers of the planned and actual implant platforms.
- 3D Deviation at the implant apex: The linear distance (mm) between the centers of the planned and actual implant apices.
- Depth deviation: The vertical discrepancy (mm) along the longitudinal axis of the implant.
- Angular deviation: The three-dimensional angle (degrees) formed between the longitudinal axes of the planned and actual implants.

### 2.6. Statistical Analysis

Descriptive statistics were calculated for all evaluated parameters, expressing data as means with standard deviations (SD), medians with interquartile ranges (IQRs), and minimum/maximum values.

The normality of the data distribution was assessed for each study group using the Shapiro–Wilk test and homogeneity of variances using Levene’s test. Intergroup comparisons were performed using one-way ANOVA when parametric assumptions were satisfied and the Kruskal–Wallis test for non-parametric distributions.

Effect sizes were interpreted according to established thresholds: for ANOVA-derived eta squared (η^2^), 0.01 was considered small, 0.06 moderate, and ≥0.14 large. Post hoc pairwise comparisons were conducted using Mann–Whitney U tests with Holm–Bonferroni correction, with effect size expressed as rank-biserial correlation (r), interpreted as small (0.10), moderate (0.30), and large (≥0.50). Statistical significance was set at *p* < 0.05. Statistical analysis was performed using MedCalc^®^ Statistical Software version 20.023 (MedCalc Software Ltd., Ostend, Belgium).

## 3. Results

No intraoperative complications were observed during the surgical procedures, and all 180 implants successfully underwent the postoperative assessment and were included in the final analysis.

### 3.1. Descriptive Analysis

Descriptive statistics for all evaluated parameters—angular deviation, 3D deviations at the neck and apex, and depth deviation—are summarized in Table 1. The data demonstrated a consistent and progressive increase in deviation values moving from tooth-supported to bone-supported, and finally to mucosa-supported surgical guides.

Tooth-supported guides exhibited the highest precision across all categories. The mean angular deviation for this group was 1.81° ± 0.45°, while linear 3D deviations at the neck and apex were 0.59 ± 0.18 mm and 0.73 ± 0.19 mm, respectively. In contrast, mucosa-supported guides showed the greatest discrepancies, with a mean angular deviation of 2.95° ± 0.60° and linear 3D deviations reaching 1.47 ± 0.29 mm at the neck and 1.87 ± 0.37 mm at the apex. Bone-supported guides occupied an intermediate position, demonstrating mean values of 2.14° ± 0.48° for angular deviation, 1.04 ± 0.26 mm at the neck, and 1.61 ± 0.31 mm at the apex.

### 3.2. Intergroup Comparisons and Effect Sizes

The results of the overall intergroup comparisons are presented in Table 2.

One-way ANOVA revealed significant differences among the three guide types for angular deviation, 3D neck deviation, and 3D apex deviation (*p* < 0.001 for all). The calculated effect sizes for these parameters were considered as large (angular: η^2^ = 0.468; neck: η^2^ = 0.679; apex: η^2^ = 0.733), indicating that the type of guide support explained a substantial portion of the total variance in implant positioning accuracy.

Regarding depth deviation, the Kruskal–Wallis test also confirmed statistically significant differences across the groups (*p* < 0.001). However, the effect size was notably smaller (ε^2^ = 0.09) compared to angular and horizontal parameters. This suggests that vertical control is relatively more stable across different support modalities than horizontal and angular orientation.

### 3.3. Post Hoc Pairwise Comparisons

Pairwise comparisons using the Mann–Whitney U test with Holm–Bonferroni correction identified specific significant differences between all guide support types for most variables, all of which remained statistically significant (*p* < 0.05) following the correction (Table 3). The exceptionally large effect sizes observed (e.g., r = 0.999) likely reflect the biomechanical contrast between the rigid, mechanical friction fit of tooth-supported guides and the highly resilient nature of mucosa-supported guides, amplified by the highly controlled, single-surgeon setting of this study.

The most pronounced differences were observed between tooth-supported and mucosa-supported guides, with large effect sizes for 3D neck deviation (r = 0.994) and 3D apex deviation (r = 0.999).

For angular deviation, all pairwise comparisons reached statistical significance, with the largest effect size found in the tooth-supported vs. mucosa-supported comparison (r = 0.869). In terms of depth deviation, significant differences were identified between tooth-supported and mucosa-supported guides (*p* < 0.001, r = 0.417) and between bone-supported and mucosa-supported guides (*p* = 0.005, r = 0.315). Notably, the difference in depth deviation between tooth-supported and bone-supported guides did not reach statistical significance (*p* = 0.182).

### 3.4. Ditribution Analysis

**Figure 4:**
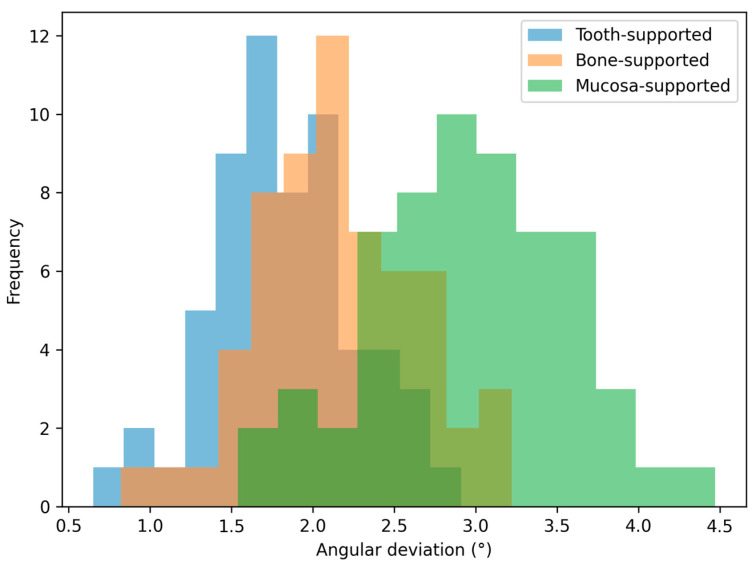
Distribution histogram for angular deviations across the three study groups.

**Figure 5:**
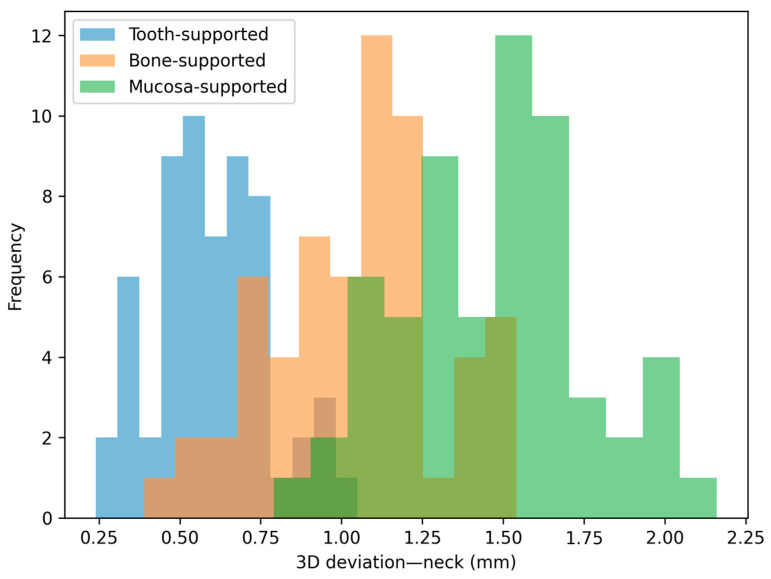
Distribution histogram for 3D linear deviations at the neck of the implant across the three study groups.

**Figure 6:**
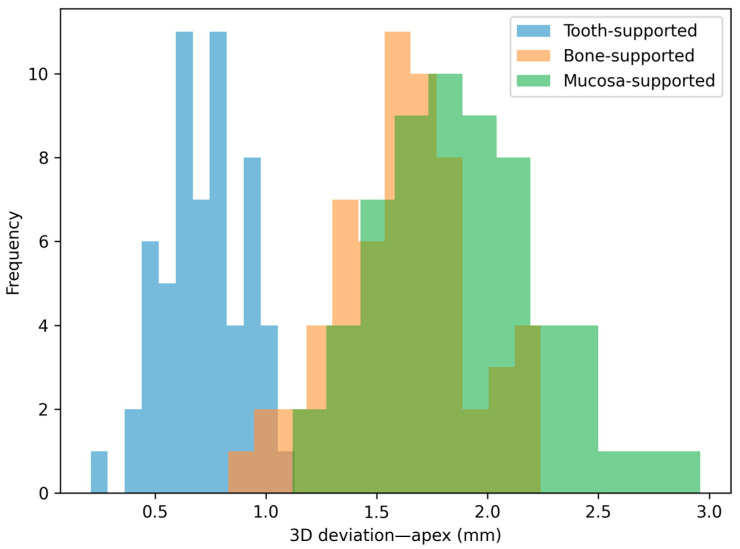
Distribution histogram for 3D linear deviations at the implant apex across the three study groups.

**Figure 7:**
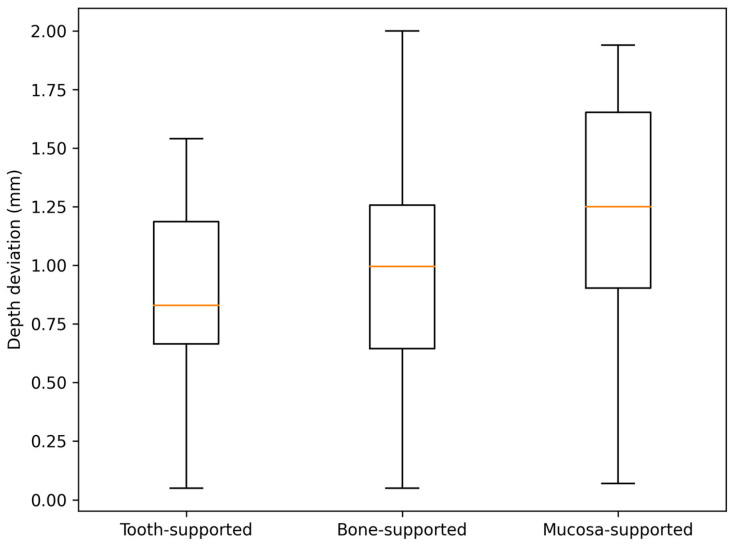
Box plot representation of depth deviations across the three study groups.

The distribution patterns of the deviations are illustrated in Figure 4, Figure 5, Figure 6 and Figure 7. Histograms for angular and horizontal deviations clearly show a rightward shift for mucosa-supported guides, statistically corresponding to the significantly higher frequency of larger errors reported in Table 1. The box plot for depth deviation (Figure 7) highlights a more uniform distribution and smaller intergroup dispersion, which is consistent with the significantly lower effect size (ε^2^ = 0.09) reported for vertical control across all support modalities.

## 4. Discussion

The main objective of this clinical study was to evaluate the influence of different guide support modalities on the accuracy of s-CAIS. The findings demonstrated that the type of guide support is a significant determinant of surgical precision. Specifically, a clear hierarchy of accuracy was established: tooth-supported guides exhibited the highest level of precision, followed by bone-supported guides, while mucosa-supported guides demonstrated the greatest linear and angular discrepancies. These results were supported by highly significant intergroup differences and large effect sizes, particularly for angular and horizontal deviations. The statistical power of this investigation provides valuable comparative data for evaluating the limitations of current digital workflows in both partially and completely edentulous patients.

The results of the present study confirm that tooth-supported surgical guides achieve the highest level of accuracy, contributing to their status as the clinical gold standard for s-CAIS. The mean angular deviation of 1.81° ± 0.45° and 3D linear deviation at the implant neck of 0.59 ± 0.18 mm observed in this group are consistent with the findings of Shi et al. [11], who reported that bilateral tooth support provides the most stable and predictable guide seat, effectively neutralizing rotational and tipping forces during the osteotomy. Moreover, these values are lower than the pooled mean values reported in the meta-analysis by Tahmaseb et al. [3], where entries for s-CAIS averaged 1.12 mm. Gallardo et al. [10] further demonstrated that tooth-supported guides significantly outperform bone- and mucosa-supported counterparts, primarily due to the anatomical stability of the remaining dentition. These findings are further reinforced by El Kholy et al. [15], who noted that the rigid seat provided by teeth minimizes intraoperative guide micro-movement, which is often the main source of angular error in edentulous cases. Most recent literature reports comparable clinical outcomes, suggesting that when stable dental reference points are utilized, the error margin between the virtual plan and clinical reality is minimized [16].

In contrast to the high precision of tooth-supported guides, edentulous scenarios present significant challenges due to the lack of stable anatomical landmarks and the inherent characteristics of the supporting tissues. The increased discrepancies observed in the mucosa-supported cohort, with a mean angular deviation of 2.95° ± 0.60° and apical deviation of 1.87 ± 0.37 mm, reflect the limitations of relying on resilient tissues, such as oral mucosa. These results align with the previously mentioned systematic review by Shi et al. [11], which identified mucosa-supported guides as having the lowest in vivo accuracy, primarily due to the “sinking” effect and mucosal displacement under vertical surgical pressure. This phenomenon is further confirmed by Cassetta et al. [17], who emphasized that the variability in mucosal thickness and its compressibility are primary determinants of guide instability, leading to significant linear and angular errors. Regarding bone-supported guides, the presented findings are markedly more precise than the results obtained by Arisan et al. [18], who reported substantially higher deviations (5.0° angular and 1.70 mm coronal) for bone-supported protocols. The results of the present study for bone-supported guides are more consistent with the observations of Vercruyssen et al. [19], who demonstrated that direct bone contact provides a more rigid and predictable foundation than mucosa, despite the surgical need of flap reflection. Chen et al. [20] suggest that the complexity of full-thickness mucoperiosteal flap elevation and repositioning of the surgical guide can introduce cumulative transfer errors, resulting in higher deviations compared to mucosa-supported flapless protocols. Furthermore, as highlighted by Azevedo et al. [7], apical discrepancies in edentulous patients consistently exceed coronal ones, which is confirmed by the presented results, indicating that the guide sleeve effectively constrains the implant platform, but the apex remains susceptible to deviations along the path of least resistance in the alveolar bone.

A notable finding in the present study is the relative consistency of depth deviation across all three groups, as evidenced by a low effect size (ε^2^ = 0.09). This suggests that vertical control is less influenced by the guide support modality than angular or horizontal parameters. This observation is supported by Vinci et al. [21], which indicated that vertical discrepancies in edentulous cases typically present the narrowest margin of error due to the standardized vertical constraints of the s-CAIS workflow. Kernen et al. [22] highlighted that the precision of virtual planning software and the standardization of guide design, specifically the sleeve-to-implant offset and physical stop mechanisms, are the primary determinants of vertical accuracy. In a meta-analysis by Yogui et al. [23], static guidance was shown to provide superior vertical predictability compared to freehand methods, as the drilling sequence is mechanically constrained by the guide’s hardware, thereby neutralizing the potential “sinking” effect. These findings imply that the risk of vertical over-preparation is effectively mitigated by the mechanical limits of the surgical kit (physical stops) rather than the biological stability of the guide’s seat. On the other hand, angular and horizontal parameters are more sensitive to the stabilization of the surgical guide.

The clinical implications derived from this study highlight the necessity of implementing rigorous safety protocols, particularly in full-arch rehabilitation. While the mean deviations observed in all groups remain within manageable limits, the maximum apical deviation of 1.87 mm recorded in the mucosa-supported group underscores the requirement for a minimum safety buffer of at least 2 mm from vital anatomical structures [24]. Garcia et al. [25] confirmed this by highlighting that such safety margins are critical to avoid permanent injury to the inferior alveolar nerve, especially in cases with reduced vertical bone height. Another factor that makes a predefined safety margin mandatory is lack of direct bone visibility in flapless mucosa-supported procedures, which was noted by Valente et al. [26]. The recent literature suggests that a 2 mm margin provides a statistically significant buffer against cortical perforations and Schneiderian membrane violations [27]. Finally, Younes et al. [28] demonstrated in a randomized controlled trial that cumulative errors, even when handled by experienced clinicians, can occasionally exceed 1.5 mm. This can increase the need for a conservative surgical approach in fully guided workflows. Consequently, the selection of the guide support modality should directly dictate the preoperative risk assessment, with mucosa-supported guides demanding the most extensive safety considerations to ensure patient safety.

The primary strength of this study lies in its sample size of 180 implants, which provides substantial statistical power to evaluate the differences between the three support modalities. This methodology expands upon our previous research by providing a direct comparison of all three support systems under a uniform measurement protocol. However, several limitations must be acknowledged. First, the retrospective design may introduce characteristic selection biases. Second, the CBCT voxel size of 160 µm, while in accordance with clinical standards, imposes a resolution limit on the superimposition process. As noted by Flügge et al. [29], the precision of the ICP algorithm is partially dependent on the quality of the volumetric data and the chosen segmentation thresholds. Third, this study did not quantify biological variables such as mucosal thickness or localized bone density. According to Tözüm et al. [30], variations in bone mineral density can influence the trajectory of the initial osteotomy, potentially affecting final accuracy. Furthermore, it is critical to acknowledge that this study compared not only different tissue support modalities but also their inherently distinct stabilization protocols. While tooth-supported guides achieved stability through a 0.10 mm friction fit on the remaining dentition, the bone- and mucosa-supported guides required horizontal anchor pins for rigid fixation. Consequently, the observed discrepancies in accuracy likely reflect a combination of the tissue support type and the specific fixation method utilized. Similarly, the digital planning workflows differed between the groups. The partially edentulous group relied on a point-to-point surface-matching registration of intraoral STL scans onto CBCT data, while the completely edentulous groups required a dual-scan protocol utilizing a radiographic guide. Because these separate digital workflows possess different inherent error margins, they act as an additional variable that may have influenced the final accuracy outcomes. While the performance of all surgical procedures by a single specialist eliminated inter-operator variability and ensured high internal consistency, this design may limit the external validity of the findings when applied to clinicians with varying levels of experience or in different clinical environments. Finally, the lack of adjustment for patient-level clustering in the statistical analysis should be considered when interpreting the reported *p*-values and effect sizes. Additionally, while the recorded deviations are within clinically acceptable ranges of at least 2 mm from vital structures [24], prospective long-term trials are necessary to determine the direct impact of these discrepancies on the functional and biological success of prosthetic rehabilitation.

## 5. Conclusions

Within the limitations of this study, it can be concluded that the type of surgical guide support is a primary determinant of implant placement accuracy. Tooth-supported guides offer the highest level of precision in s-CAIS. In edentulous cases, bone-supported guides demonstrated higher angular and linear accuracy compared to mucosa-supported guides. However, regardless of the support modality, vertical depth remains the most stable and predictable parameter. In conclusion, the choice of guide support should be integrated into a preoperative risk assessment. When utilizing mucosa-supported guides, clinicians must strictly adhere to wider safety margins to mitigate the risks associated with tissue displacement.

## Figures and Tables

**Table 1:** Descriptive statistics of deviation parameters according to guide type.

Variable	Guide Type	Mean ± SD	95% Confidence Intervals (CI)	Median (IQR)	Min–Max
Angular deviation (°)	Tooth-supported	1.81 ± 0.45	1.70–1.92	1.77 (0.48)	0.65–2.91
Angular deviation (°)	Bone-supported	2.14 ± 0.48	2.02–2.26	2.09 (0.61)	0.82–3.22
Angular deviation (°)	Mucosa-supported	2.95 ± 0.60	2.80–3.10	2.92 (0.80)	1.54–4.47
3D deviation—neck (mm)	Tooth-supported	0.59 ± 0.18	0.54–0.64	0.58 (0.22)	0.24–1.05
3D deviation—neck (mm)	Bone-supported	1.04 ± 0.26	0.97–1.11	1.08 (0.33)	0.39–1.54
3D deviation—neck (mm)	Mucosa-supported	1.47 ± 0.29	1.40–1.54	1.51 (0.37)	0.79–2.16
3D deviation—apex (mm)	Tooth-supported	0.73 ± 0.19	0.68–0.78	0.72 (0.23)	0.21–1.13
3D deviation—apex (mm)	Bone-supported	1.61 ± 0.31	1.53–1.69	1.62 (0.36)	0.83–2.24
3D deviation—apex (mm)	Mucosa-supported	1.87 ± 0.37	1.78–1.96	1.86 (0.51)	1.12–2.96
Depth deviation (mm)	Tooth-supported	0.87 ± 0.39	0.77–0.97	0.83 (0.52)	0.05–1.54
Depth deviation (mm)	Bone-supported	0.97 ± 0.44	0.86–1.08	0.99 (0.61)	0.05–2.00
Depth deviation (mm)	Mucosa-supported	1.21 ± 0.46	1.09–1.33	1.25 (0.75)	0.07–1.94

**Table 2:** Intergroup comparisons of evaluated deviation parameters and calculated effect sizes.

Variable	Test	Effect Size	*p*-Value
Angular deviation (°)	ANOVA	η^2^ = 0.468	5.15 × 10^−25^
3D deviation—neck (mm)	ANOVA	η^2^ = 0.679	2.12 × 10^−44^
3D deviation—apex (mm)	ANOVA	η^2^ = 0.733	1.97 × 10^−51^
Depth deviation (mm)	Kruskal–Wallis	ε^2^ = 0.09	1.63 × 10^−4^

**Table 3:** Post hoc pairwise comparisons between different guide support types with Holm–Bonferroni correction.

Variable	Comparison	Test	Adjusted *p*-Value	Effect Size (r)
Angular deviation (°)	Tooth-supported vs. Bone-supported	Mann–Whitney U	1.22 × 10^−4^	0.407
Angular deviation (°)	Tooth-supported vs. Mucosa-supported	Mann–Whitney U	6.67 × 10^−16^	0.869
Angular deviation (°)	Bone-supported vs. Mucosa-supported	Mann–Whitney U	5.25 × 10^−11^	0.706
3D deviation—neck (mm)	Tooth-supported vs. Bone-supported	Mann–Whitney U	8.81 × 10^−15^	0.830
3D deviation—neck (mm)	Tooth-supported vs. Mucosa-supported	Mann–Whitney U	1.88 × 10^−20^	0.994
3D deviation—neck (mm)	Bone-supported vs. Mucosa-supported	Mann–Whitney U	8.07 × 10^−12^	0.724
3D deviation—apex (mm)	Tooth-supported vs. Bone-supported	Mann–Whitney U	2.11 × 10^−20^	0.988
3D deviation—apex (mm)	Tooth-supported vs. Mucosa-supported	Mann–Whitney U	1.11 × 10^−20^	0.999
3D deviation—apex (mm)	Bone-supported vs. Mucosa-supported	Mann–Whitney U	1.08 × 10^−4^	0.410
Depth deviation (mm)	Tooth-supported vs. Bone-supported	Mann–Whitney U	1.82 × 10^−1^	0.141
Depth deviation (mm)	Tooth-supported vs. Mucosa-supported	Mann–Whitney U	2.50 × 10^−4^	0.417
Depth deviation (mm)	Bone-supported vs. Mucosa-supported	Mann–Whitney U	5.89 × 10^−3^	0.315

## Data Availability

The original contributions presented in the study are included in the article, further inquiries can be directed to the corresponding author.
